# Multiscale Strain Transfer in Cartilage

**DOI:** 10.3389/fcell.2022.795522

**Published:** 2022-02-04

**Authors:** Manuela A. Boos, Shireen R. Lamandé, Kathryn S. Stok

**Affiliations:** ^1^ Department of Biomedical Engineering, The University of Melbourne, Parkville, VIC, Australia; ^2^ Musculoskeletal Research, Murdoch Children’s Research Institute, Parkville, VIC, Australia; ^3^ Department of Paediatrics, The University of Melbourne, Parkville, VIC, Australia

**Keywords:** cartilage, chondrocytes, mechanotransduction, tissue strain, ECM, heterogeneity

## Abstract

The transfer of stress and strain signals between the extracellular matrix (ECM) and cells is crucial for biochemical and biomechanical cues that are required for tissue morphogenesis, differentiation, growth, and homeostasis. In cartilage tissue, the heterogeneity in spatial variation of ECM molecules leads to a depth-dependent non-uniform strain transfer and alters the magnitude of forces sensed by cells in articular and fibrocartilage, influencing chondrocyte metabolism and biochemical response. It is not fully established how these nonuniform forces ultimately influence cartilage health, maintenance, and integrity. To comprehend tissue remodelling in health and disease, it is fundamental to investigate how these forces, the ECM, and cells interrelate. However, not much is known about the relationship between applied mechanical stimulus and resulting spatial variations in magnitude and sense of mechanical stimuli within the chondrocyte’s microenvironment. Investigating multiscale strain transfer and hierarchical structure-function relationships in cartilage is key to unravelling how cells receive signals and how they are transformed into biosynthetic responses. Therefore, this article first reviews different cartilage types and chondrocyte mechanosensing. Following this, multiscale strain transfer through cartilage tissue and the involvement of individual ECM components are discussed. Finally, insights to further understand multiscale strain transfer in cartilage are outlined.

## Introduction

All tissues in the body contain cells and a well organised extracellular matrix (ECM) compartment. The ECM is tissue-specific and the constituents, such as collagens, proteoglycans (PGs), and elastin, vary between different tissues. The ECM provides physical support and scaffolding for the cells, and also regulates crucial biochemical and biomechanical cues that are required for tissue morphogenesis, differentiation, growth, and homeostasis (Frantz et al. 2010; Theocharis et al. 2016). The cells within these tissues establish the ECM during development, maintain it in healthy tissue, and repair it in response to disease and injury (Lu et al. 2011; Humphrey et al. 2014). This reciprocal relationship between the cells and the ECM is based on the ability of cells to sense physical signals and transduce them into biochemical responses. Converting mechanical signals into chemical signals is called mechanotransduction (Mofrad et al. 2010; Wang 2017). Cross talk between cells and ECM creates a local environment where matrix stiffness and the physical forces sensed by the cells play an essential role in biological functions of cell and tissue physiology, and lead to constant tissue remodelling. Given the finite life span of cells and ECM components, this remodelling ensures a homeostatic balance in the tissue, e.g. structural integrity and functionality. This homeostasis is thus achieved by balanced matrix degradation and deposition of new constituents. Disruption of these homeostatic processes leads to tissue degeneration, fibrosis or other pathologies.

Cartilage is a connective tissue providing mechanical and structural support in different anatomical locations in the human body. Its ECM is produced by the relatively scarce specialised cells, chondrocytes, and is mainly composed of PGs, different collagen types, and elastin. It is classified into three different types, hyaline cartilage (articular joints, nose, ribs), elastic cartilage (ears, larynx), and fibrocartilage (menisci, intervertebral discs). These different types vary in their histological and physiological appearance and also in the magnitude of physical daily load they experience (Homicz et al. 2003; Heinegard et al. 2011; McNulty et al. 2015; Nimeskern et al. 2015). Furthermore, different cartilage types have a distinct ECM composition, with a highly heterogeneous accumulation of different proteins, molecules, and fibres. This variation in tissue composition between cartilage types leads to heterogeneous transfer of mechanical stimuli and influences cell morphology and biochemical response. Our knowledge about the mechanoresponse of cartilage has tremendously increased. Single cell responses to mechanical stimuli are well studied in health and disease. However, mechanical signals do not originate in the immediate vicinity of the cells. Rather they result from movements of the whole body and forces from body weight and joint movement. Even though we know well how chondrocytes respond to mechanical stimuli, we do not fully understand how these signals reach the cells, as it is a multiscale process. Furthermore, it is not well known how different ECM compositions and arrangements influence load transfer to cells. Specifically, how heterogeneous strain in the ECM influences chondrocytes and therefore long-term remodelling of cartilage in both health and disease, is not known.

Understanding these processes and mechanisms is as critical to progress in tissue regeneration and repair strategies as it is to engineering cartilage tissues. Gaining further insights into multiscale strain transfer and mechanotransduction in different cartilage types would provide a benchmark by which to compare tissue engineered constructs, and feed into developing effective treatment strategies to address cartilage pathologies. Therefore, this article first reviews different cartilage types and chondrocyte mechanosensing. Following this, multiscale strain transfer through cartilage tissue and the involvement of individual ECM components are discussed. Finally, insights to further understand multiscale strain transfer in cartilage are outlined.

## Different Cartilage Types

### Hyaline Cartilage

In hyaline cartilage, the so-called ‘solid’ phase of the ECM is mainly composed of collagen II (15%–22%) and PGs (4%–7%). The PGs are comprised of different glycosaminoglycans (GAG) chains attached to a core protein. The fluid phase on the other hand consists of up to 80% water (Eyre 2002; Heinegard 2009; Sophia Fox et al. 2009). This water content is a result of the fixed charge density created by the negatively charged sulphated GAGs. Their charge attracts cations which leads to osmotic pressure in the tissue. As the PGs with the attached GAGs swell, they are physically limited by the collagen network which gives articular cartilage a high compressive resilience. This feature is further increased under physical load as the repulsive forces of the PGs are pushed closer together and swelling is hindered by the tension in the collagen fibres (Roth et al. 1980). This effect enables articular cartilage to withstand significant loads up to multiple times body weight (Mansour, 2003; Lu et al. 2009; Sophia Fox et al. 2009; Thielen et al. 2019). The experienced physical load of the cartilage varies depending on anatomical location.

**FIGURE 1:**
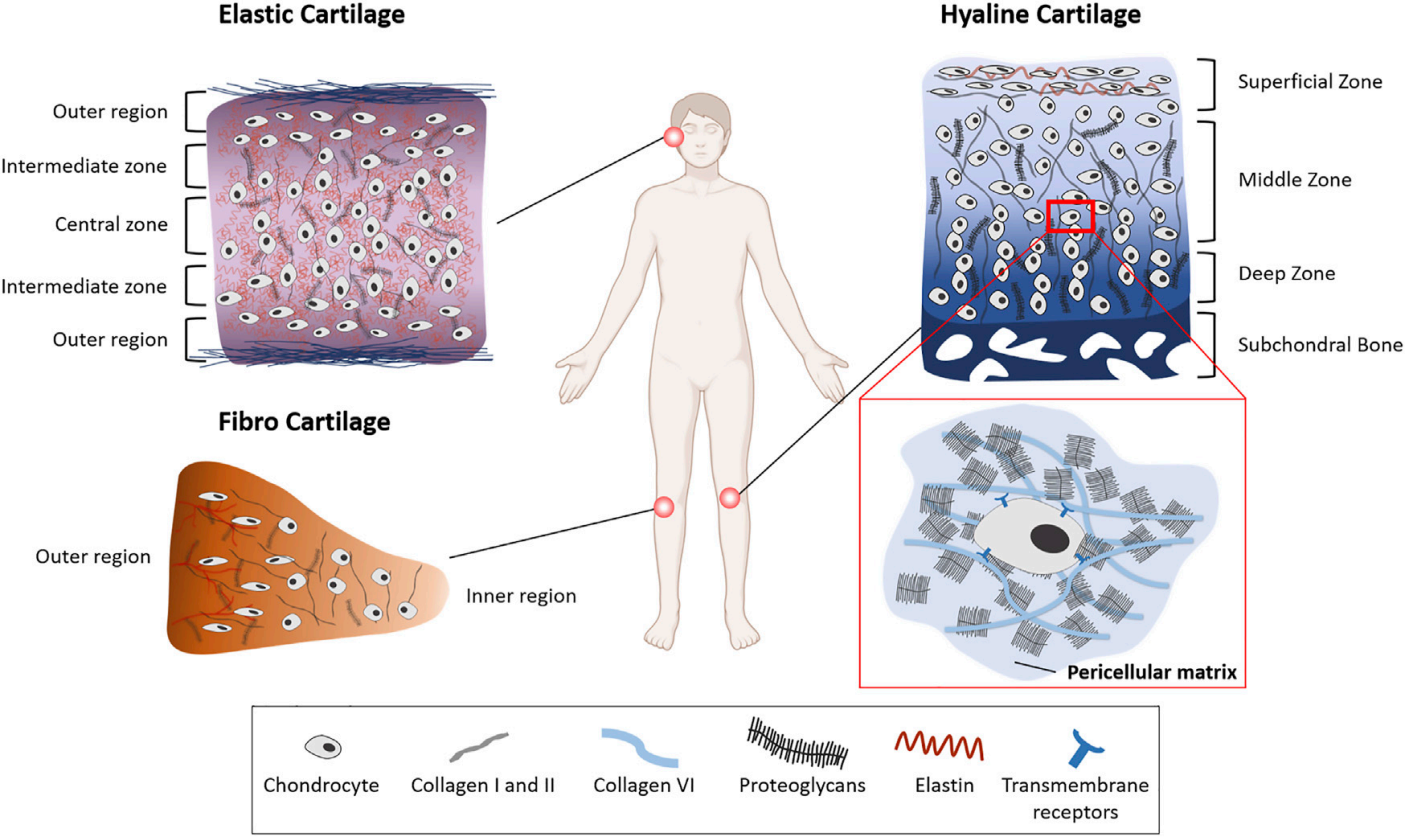
Extracellular matrix heterogeneity of different cartilage types. The three cartilage types vary in ECM composition and arrangement. Elastic cartilage and hyaline articular cartilage are arranged in different zones with the chondrocyte shape becoming smaller and flatter towards the outer region and superficial zone, respectively. Proteoglycan (PG) concentration articular cartilage in the knee joint increases with depth of the tissue. In meniscal cartilage (fibro cartilage) the PG concentration is highest in the outer region. The pericellular matrix (PCM) surrounds the chondrocytes and contains a high amount of PGs such as aggrecan and perlecan, and collagen type VI in articular and meniscal cartilage.

Articular cartilage is divided into a superficial zone, a middle zone, and a deep zone, which vary in their ECM composition, cell orientation and morphology (Figure 1). In the superficial zone chondrocytes are more abundant and have a flattened, elongated form compared to the deeper zones where they are less densely populated. In deeper zones the chondrocytes are more spherical and are arranged in columns. The PG content in articular cartilage increases with depth. The collagen content however remains constant with increasing depth, but fibres change from being arranged parallel to the surface in the superficial zone to a perpendicular orientation in the deeper zones (Eggli et al. 1988; Guilak, 1995; Wong et al. 1996; Poole et al. 2001; Athanasiou et al. 2009; Quinn et al. 2013). Elastin is present in the superficial zone of articular cartilage (Mansfield et al. 2009). The elastin fibres run parallel to the surface and roughly in the same direction as the collagen fibres (Yeh et al. 2005; Yu et al. 2010).

All chondrocytes in the tissue are surrounded by a PG-rich pericellular matrix (PCM) (Figure 1) (Poole 1997). The PCM has a crucial function in absorbing, redistributing, and transmitting mechanical forces in articular and meniscal cartilage (Poole 1997; Sanchez-Adams et al. 2013; Gilbert et al. 2018) (Millward-Sadler et al. 2004). It shields chondrocytes from extensive stress, and because it is a direct link between the cells and the ECM makes an important contribution to transmitting biomechanical signals to the cells (Quinn et al. 1998). The PCM has a high concentration of aggrecan and other PGs such as perlecan, biglycan and decorin which play an important role in its integrity (Gomes et al. 2002; Vincent et al. 2007; Wilusz et al. 2014; Shu et al. 2016). In addition to PGs, collagen type VI in articular cartilage is highly concentrated in the PCM and plays an important role in its mechanical integrity. It connects the chondrocytes to the PCM through β-integrin receptors and transmembrane PGs (Marcelino et al. 1995; Wilusz et al. 2012). The PCM in the articular cartilage superficial zone also contains elastin and lipids (Poole 1997; Mansfield et al. 2009). As the tensile and shear stresses are higher in this zone, this composition would protect the chondrocytes from these stresses.

### Fibrocartilage

Located in the knee joint, the menisci, composed of fibrocartilage (Figure 1), are responsible for increasing the contact area and distributing forces across the joint. In contrast to hyaline and elastic cartilage, the main collagen type in the menisci is type I (Fox et al. 2015). Collagen fibres are oriented circumferentially in the interior layers of meniscal cartilage compared to radially oriented fibres in the outer regions (Amis 2010). The circumferential alignment of these collagen fibres contributes to the load bearing properties of the menisci by converting compressive axial stresses to tensile hoop stresses (McDermott et al. 2008; Sanchez-Adams et al. 2013; Fox et al. 2015). Like articular chondrocytes, cells in meniscal cartilage differ in their appearance depending on their location within the tissue. Cells in the outer region of meniscal cartilage have an oval, fusiform shape, whereas cells in the inner region have a round shape (Hellio Le Graverand et al. 2001; Makris et al. 2011). In contrast to the large amount of collagen type I in outer regions of the meniscus, the inner region has more collagen type II (60%) than type I (40%) (McDermott, 2010; Sanchez-Adams et al. 2013). PG-rich regions are interspersed between these collagen fibres, leading to a highly inhomogeneous tissue (Upton et al. 2008; Han et al. 2013; Han et al. 2016). Similarly to the PCM in hyaline cartilage, the PCM in meniscal cartilage is mainly composed of perlecan and collagen type VI (Sanchez-Adams et al. 2013) (Figure 1).

### Elastic Cartilage

Elastic cartilage is not exposed to large biomechanical forces, as it is found in the head and neck region. It contains a high amount of elastin which is arranged around the chondrocytes and contributes to maintaining anatomic shape *via* complex heterogeneous arrangement of tensile compressive fibre networks (Figure 1) (Culav et al. 1999; He et al. 2013; Nimeskern et al. 2016). Elastic ear cartilage is arranged in different zones as well. Similar to articular cartilage, the chondrocytes in the outer regions are smaller and flatter than the cells in the intermediate and central zones, where the chondrocytes are larger and further apart (Keith et al. 1977; Jessop et al. 2016). Collagen type II in auricular cartilage is arranged together with a dense elastin fibre network surrounding the chondrocytes in a honeycomb like structure (Chen et al. 2014; Bos et al. 2018). The composition of the PCM of elastic cartilage has not been reported.

Despite their differences in ECM structure, all three cartilage types are avascular (except the outer region of the menisci), aneural, and alymphatic, resulting in a tissue with limited intrinsic repair capabilities (Khan et al. 2008; Makris et al. 2011). These variations in the cartilage ECM make it a highly heterogeneous tissue on a macrostructural as well as microstructural level. These structural differences in cartilage types directly influence the mechanical environments experienced by the chondrocytes and this heterogeneity leads to nonuniform strain and stress in cartilage. This in turn, changes how mechanical signals are transmitted and received by the cells in these different tissues.

## Chondrocyte Mechanosensing

**FIGURE 2:**
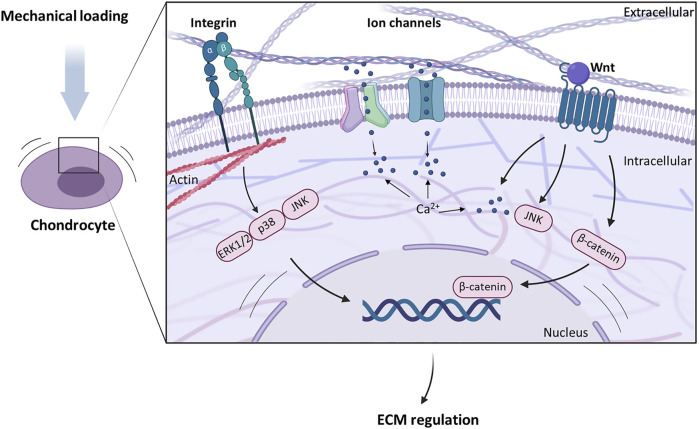
Different cell membrane receptors and signalling molecules are involved in chondrocyte mechanotransduction. Transmembrane ion channels induce intracellular Ca^2+^ signalling upon mechanical stimulation of cartilage and regulate extracellular matrix (ECM) biosynthesis. Integrins serve as a link between ECM molecules and the cytoskeleton and lead to an activation of the MAPK pathway. Signalling molecules such as Wnt activate intracellular signalling cascades that are important for cartilage homeostasis.

Chondrocytes establish the cartilage ECM during development and maintain it in healthy tissue (Lu et al. 2011 *;* Humphrey et al. 2014). This reciprocal relationship between the chondrocytes and ECM is based on their ability to sense physical signals and transduce them into biochemical responses, making them highly mechanosensitive. This conversion of mechanical signals into chemical signals is called mechanotransduction (Mofrad et al. 2010 *;* Wang 2017) and this allows chondrocytes to sense changes in ECM properties. The response of articular chondrocytes to mechanical stimuli is well studied. They have several cell surface mechanoreceptors, e.g. ion channels, integrin receptors, and primary cilia, that are sensitive to changes in intrinsic tissue stiffness and external tissue compression (Figure 2) and initiate intracellular signalling cascades that modulate gene expression leading to ECM remodelling (Gilbert et al. 2018). Furthermore, intracellular deformation and signalling molecules enable responses to changes in mechanical environment.

### Ion Channels

Ion channels such as transient receptor potential vanilloid 4 (TRPV4) and Piezo channels are Ca^2+^-permeable and situated in the plasma membrane where they play a role in mechanically induced Ca^2+^ signalling. Mechanical perturbations such as changes in membrane tension, lipid bilayer distortion and osmotic stress activate these channels. This facilitates Ca^2+^ influx into the chondrocyte and initiates intracellular signalling pathways (Guilak et al. 1999; Pingguan-Murphy et al. 2006). TRPV4 is highly expressed in chondrocytes and promotes anabolic responses. It provides an essential link between mechanical loading and ECM synthesis (Phan et al. 2009; O'Conor et al. 2014; Zelenski et al. 2015). Piezo 1 and 2 channels however are involved in transducing hyperphysiological mechanical stimuli resulting from injuries or overload (Lee et al. 2014; Lee et al. 2017). Mechanical integrity of the ECM and mechanical stimuli experienced by the cells are therefore tightly related to the resulting cellular response.

### Integrins

Integrins also play an important role in chondrocyte mechanosensing. They are a family of transmembrane proteins comprised of α and β subunits. Their large extracellular domains bind to PCM ligands such as fibronectin and collagen type VI, whereas the cytoplasmic domains bind to the actin skeleton of the chondrocytes, making integrins a transmembrane link between ECM molecules and the cytoskeleton (Millward-Sadler et al. 2000; Hynes 2002; Wolfenson et al. 2013). Mechanical stimulation activates integrins, and also increases their expression in chondrocytes (Lucchinetti et al. 2004). Furthermore, cell death resulting from cartilage injuries and overload has been associated with integrin-mediated signalling. Integrins transduce mechanical signals from the ECM to the chondrocytes and are sensitive to changes in mechanical properties and stimuli.

The activation of integrins can result in activation and phosphorylation of mitogen-activated protein kinases (MAPKs). MAPKs comprise ERK1/2, p38 and JNK (Fitzgerald et al. 2008). Their activation leads to many intracellular processes like cell division, differentiation, apoptosis, and transcription. A shear strain of 3% applied at 0.1 Hz for up to 24 h has led to an activation of ERK1/2 and p38K in bovine cartilage explants. The inhibition of ERK1/2 or p38K however abolished the mechanically induced transcription of aggrecan and type II collagen (Fitzgerald et al. 2008). JNK is thought to have role in load-induced matrix anabolism. Cyclic loading at 0.33 Hz for up to 3 h induced JNK activation together with increased PG synthesis in human chondrocyte monolayers (Zhou et al. 2007).

### Primary Cilia

Primary cilia are microtubule-based structures extending from the chondrocyte surface into the PCM where they sense matrix deformation and osmotic changes through integrins in the primary cilia membrane and extracellular matrix components, and through mechanosensing ion channels. (McGlashan et al. 2006; Ruhlen et al. 2014). In chondrocytes under 15% compressive strain primary cilia were involved in upregulated PG synthesis (Wann et al. 2012). Furthermore, they have been shown to transduce mechanical signals *via* activation of the MAPK/ERK pathway. A loss of cilia leads to inhibition of downstream cartilage matrix gene expression such as type II collagen (COL-II), type X collagen (COL-X) and BMP-2 (Irianto et al. 2014). As a result, primary cilia play a crucial role in cartilage ECM formation.

### Intracellular Deformation

Cytoskeletal organisation and cell shape likewise influence chondrocyte metabolism and activate intrinsic signalling (Ingber et al. 1994; Guilak 1995). Tissue compression as well as changed mechanical properties can lead to compressive deformation of the cellular components, including the nucleus, endoplasmic reticulum, cytoskeleton, and integrins (Guilak 1995; Mobasheri et al. 2002; Wong et al. 2003; Szafranski et al. 2004). These events either lead to direct changes in gene expression or protein synthesis or induce other signalling cascades like intracellular calcium signalling (D'Andrea et al. 2000; Guilak et al. 2000; Roberts et al. 2001; Martins et al. 2012; Hall 2019).

The actin skeleton and the vimentin network are responsible for cell integrity. Disrupting actin leads to a 90% reduction in cell stiffness (Trickey et al. 2004). Following actin disruption, nuclear height and shape are severely changed, which is likely to have an effect on the biochemical response of the cells (Guilak 1995; Martins et al. 2012). Furthermore, the actin skeleton is highly sensitive to mechanical stimulation. High hydrostatic pressure (15–30 MPa) can break down the actin network, and dynamic or static loading has resulted in actin remodelling in bovine chondrocyte monolayers (Parkkinen et al. 1995). This sensitivity of actin to loading and the resulting changes in cell stiffness likely influence chondrocyte gene expression and protein biosynthesis. The vimentin network is prominent in weight-bearing areas of rabbit articular cartilage, whereas it is disassembled in unloaded rat articular cartilage explants (Eggli et al. 1988; Durrant et al. 1999). This suggests a similarly important role of vimentin in chondrocyte homeostasis (Blain et al. 2006).

### Wnt Signalling

Biomechanical effects in cartilage are mediated through activating and/or suppressing intracellular signalling pathways. Wnts are a family of signalling molecules that are essential during chondrogenesis and chondrocyte homeostasis (Tamamura et al. 2005; Yuasa et al. 2008). Wnt is bound to the ECM, mainly to heparan sulphate, a specific GAG chain (Takada et al. 2017)and its activation is thought to occur through mechanical as well as enzymatic activity (Scholtes et al. 2018; Thielen et al. 2019). The downstream effects of Wnt ligands include translocation of b-catenin to the nucleus to effect transcription, regulation of Ca^2+^ release, and cytoskeleton reorganisation. *In vivo* both excessive activation and lack of Wnt leads to cartilage breakdown, therefore the mechanoregulation of Wnt signalling is essential for cartilage integrity and homeostasis (Zhu et al. 2008; Zhu et al. 2009; Nalesso et al. 2011).

### Mechanosensing in Meniscal Cartilage

How chondrocytes in meniscal cartilage respond to mechanical forces remains largely unknown. Like articular chondrocytes, meniscal chondrocytes are highly mechanosensitive. Physiological compression of porcine *ex vivo* meniscus explants at a strain rate of 10% increases aggrecan gene expression (Aufderheide et al. 2006; Zielinska et al. 2009). However, 20% strain induces a catabolic response and increased PG breakdown (McHenry et al. 2006; Zielinska et al. 2009). Even though we have gained more insight into biochemical activities in meniscal cartilage under load, many underlying mechanisms still need to be investigated.

### Mechanosensing in Elastic Cartilage

Elastic cartilage is not exposed to constant high loading, and so, although they are mechanosensitive, mechanotransduction processes in elastic cartilage chondrocytes have not been established. When subjected to dynamic compression, porcine auricular chondrocytes upregulate collagen I, collagen II, and aggrecan gene expression (Chung et al. 2008); however, hese cells were seeded in hyaluronan hydrogels which do not mimic the native ECM components of elastic cartilage. An ECM that lacks elastin may therefore not be an ideal scaffold to study cellular response of chondrocytes from elastic cartilage. The influence of this distinct ECM in auricular cartilage on the chondrocytes remains to be investigated.

We have a good understanding of how articular chondrocytes respond to mechanical stimuli *in vitro* and which mechanosensitive channels, receptors and signalling pathways are activated upon stimulation. However, it is also essential to comprehend how stress and strain resulting from movement and body weight are transferred through the ECM to the cells *in situ*. Mechanical stimulation upregulates ECM biosynthesis and deposition. Therefore, there is likely a direct relationship between ECM composition, mechanical properties and resulting cellular response which should be further explored.

## Strain Transfer and Individual ECM Components

Body movements are complex resulting in different stresses and strains in all cartilage tissues. In the knee strains in articular cartilage can reach up to around 20% depending on the movements and are higher in more load bearing regions (Eckstein et al. 1999; Liu et al. 2010; Coleman et al. 2013). Even though compression is the main force experienced by joints, their movements also include sliding and rotation. Therefore, articular cartilage is also subjected to shear forces (Wong et al. 2008).

Meniscal cartilage is important for absorbing shock and distributes more than 50% of the axial load on the knee joint (Fukubayashi et al. 1980; Fithian et al. 1990). The strains measured in meniscal cartilage under physiological loading are in the range of 3–8% (Kolaczek et al. 2016).

Strain transfer involves organs, tissues, and cells, originating at a macro level, then travelling through the tissues to reach the cells and elicit a response. The cartilage ECM is highly heterogeneous (Figure 1) ensuring that force transfer is a complex, nonlinear and nonuniform process.

### Bulk Cartilage Tissue Strain is Heterogeneous

The heterogeneity in articular and fibrous cartilage leads to non-uniform compressive strain gradients that are depth-dependent (Eckstein et al. 1999; Erne et al. 2005; Upton et al. 2008). Articular cartilage explants exhibit distinct depth-dependent strain distributions in response to uniaxial compression. The tissue strain in the superficial layer is significantly higher compared to strain in the middle and deep zones in response to tensile and compressive forces (Erne et al. 2005; Mansfield et al. 2015). The higher compressive modulus in deeper zones of articular cartilage correlates with the higher PG content in these zones. Swelling pressure determines the tissue’s response to compression. Therefore, in the superficial zone with a lower PG concentration, water is lost more easily as the swelling pressure is lower (Chen et al. 2001).

In meniscal cartilage, strain transfer through the tissue was also heterogeneous and tissue specific (Han et al. 2013). The strain was less attenuated through the tissue at low applied strains (3%–6%) and more attenuated with higher applied strains (6%–15%).

In non-load bearing cartilage, i.e., elastic cartilage, the specific strain distribution has not been reported. Only the mechanical properties of the bulk tissue have been analysed (Nimeskern et al. 2016; Chang et al. 2020).

**FIGURE 3:**
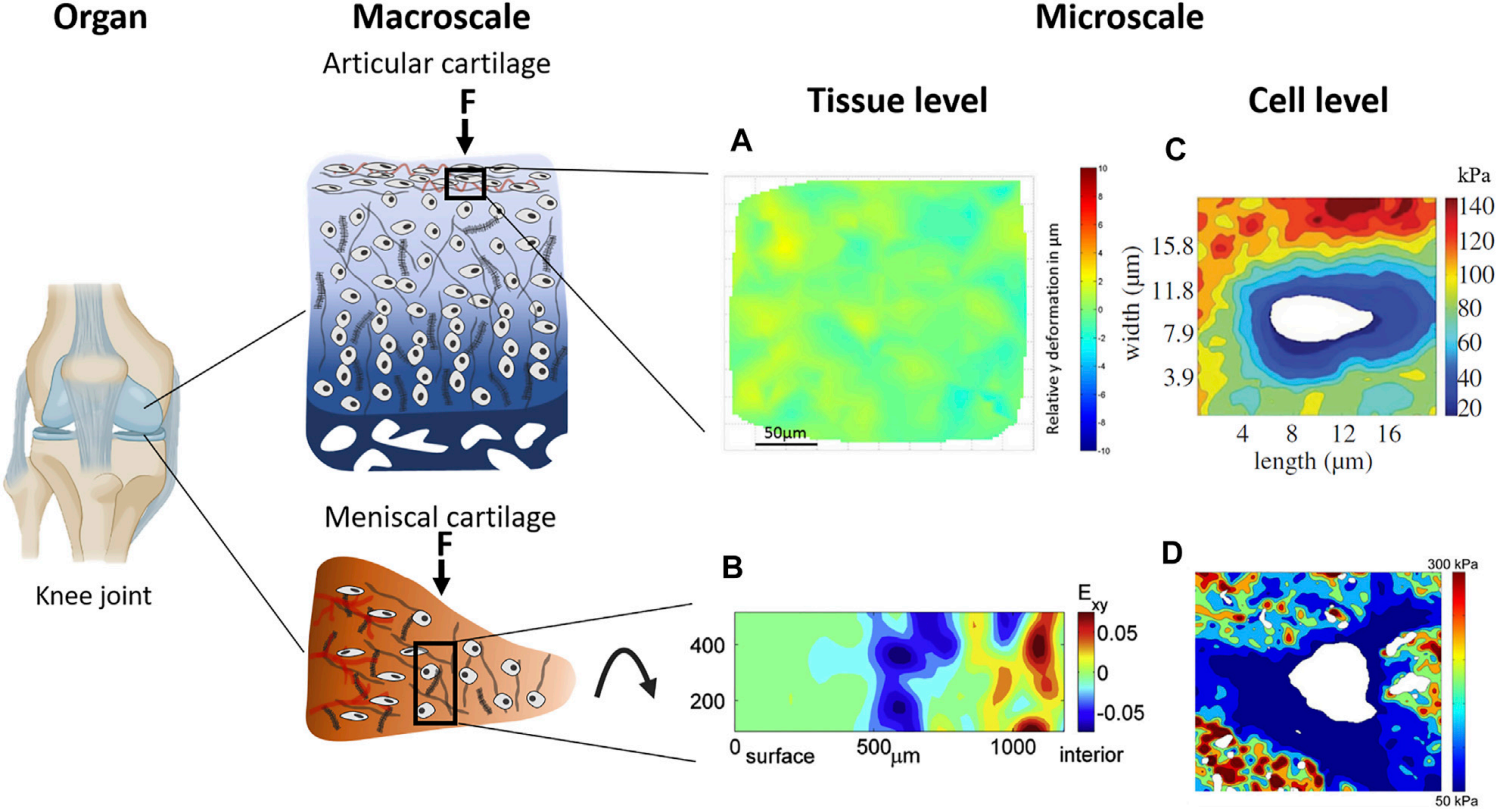
Multiscale strain transfer in articular and meniscal cartilage occurs from the organ to macroscale and microscale. Tissue strains resulting from mechanical compression and tensile loading are non-uniform. **(A)** Heterogeneous strain in the superficial zone of *ex vivo* equine articular cartilage at 38% compressive strain demonstrated by (Mansfield et al. 2015), and **(B)** heterogeneous strain distribution in the central region of *ex vivo* bovine medial meniscal cartilage at 10% compressive strain demonstrated by (Lai et al. 2010). Stiffness maps of the PCM measured with atomic force microcopy reveal a softer PCM compared to the surrounding ECM in both **(C)** the superficial zone of human articular cartilage and **(D)** the middle zone of human meniscal cartilage. Panel **(A)** from (Lai et al. 2010), **(B)** from (Han et al. 2016), **(C)** from (Wilusz et al. 2012), and **(D)** from (Sanchez-Adams et al. 2013) used with permission.

Despite our good understanding of the mechanics of different zones, the underlying mechanisms and micromechanics of the local tissue composition around the cells remain to be fully investigated. A wide range of cellular responses in the same zone of articular cartilage indicates that cells might not experience the same mechanical loading on a microscale level (Mansfield et al. 2015 *;* Kwon et al. 2016). Microscale strain is also highly heterogeneous in fibrocartilage (Figure 3) (Mansfield et al. 2015; Han et al. 2016). Looking beyond bulk tissue mechanics has revealed mechanical loading produces highly heterogeneous microscale strains potentially influencing chondrocyte responses and tissue remodelling. Investigating this local microscale environment is key to unravelling how forces from tissue scale are transmitted to cell level. Therefore, exploring the contribution of the individual ECM components and their interaction under bulk load is essential to understand the micromechanics and investigate the influence of heterogeneous strain on tissue health and disease.

### Collagen

Collagen fibres in articular cartilage have been widely explored in relation to tissue mechanics. They are able to resist very high tensile loads. Tensile modulus is largest in the direction parallel to the long axis of collagen fibres and up to 5 times higher in the superficial zone of articular cartilage compared to deeper zones (Figure 1). Tensile modulus is related to the degree of crosslinking between collagen fibres (Huang et al. 2005; Responte et al. 2007). In the superficial zone of articular cartilage, where collagen fibres are parallel to the surface, the strain following tensile load is highly heterogeneous. Tensile strain of 6% either applied along the main axis of the collagen fibres or transverse to the main collagen axis in the superficial zone resulted in heterogeneous strain distribution (Mansfield et al. 2015). This heterogeneity in the tensile strain field is thought to be due to the leaf-like organisation of the collagen layers (Nickien et al. 2013; Mansfield et al. 2015).

### Proteoglycans

Under compression, the swelling pressure resulting from the GAGs is resisted by the tensile forces of the collagen fibres. The PG concentration increases with tissue depth (Figure 1) and correlates with the compressive modulus (Mansfield et al. 2015). On a microscale level, while Irwin *et al.* observed only a weak correlation between GAG content and strain magnitude (Irwin et al. 2021) they acknowledged it is difficult to distinguish GAGs from collagen fibres using Raman spectroscopy and so the measured strain might not have resulted from GAGs solely. Furthermore, strain transfer in PG-rich regions in meniscal cartilage was significantly attenuated compared to fibre rich regions which suggests an intimate and direct involvement of the PGs in strain attenuation and distribution (Han et al. 2016). Diseases like osteoarthritis are related to PG loss and impaired mechanical tissue integrity, which leads to a cascade of deteriorating events. Therefore, a clear understanding of the role of PGs in stress and strain transfer at the microscale is important.

### Elastin

The elastin fibres in the superficial zone of articular cartilage are always under tension and a strain of around 15% even in unloaded tissue. Under compression, the fibres show minimal deformation or movement and there is no difference in compressive response when elastin is depleted from articular cartilage (Mansfield et al. 2015; Nimeskern et al. 2016). Therefore, elastic fibres are thought to be involved in recovery after shear and tensile stress (Mansfield et al. 2015). These stresses are highest in the superficial zone of articular cartilage, where the majority of the elastin fibres are present. In auricular cartilage, however, elastin is a major ECM component. Extensive mechanical evaluation has highlighted its importance in the viscoelastic response of auricular cartilage. The tissue loses its compressive and tensile integrity following elastin depletion (Nimeskern et al. 2016).

### Pericellular Matrix

The high PG concentration in the PCM produces a high negative charge which attracts counterions therefore providing immediate Ca^2+^ sources for channel activation and swelling, giving it its distinct mechanical properties. The ECM in articular cartilage outside the PCM has a stiffness of up to 1000 kPa, whereas the stiffness of the PCM is only around 50 kPa with a decrease of stiffness in towards deeper zones of the tissue (Figure 3) (Darling et al. 2010; Wilusz et al. 2012). In meniscal cartilage the PCM stiffness is around 150 kPa in the outer region gradually decreasing towards the inner region where the stiffness is around 30 kPa (Sanchez-Adams et al. 2013). The chondrocytes themselves have the lowest stiffness, which is 0.1 kPa (Alexopoulos et al. 2003; Steklov et al. 2009; Darling et al. 2010; Nia et al. 2015; Carlson et al. 2017; Chery et al. 2020).

The PCM mechanical properties vary with changes in protein and PG composition. Reduced PG content results in a less negative environment and thus less Ca^2+^ leading to reduced osmotic swelling, ultimately changing the stiffness (Guilak et al. 2006; Danalache et al. 2019; Zhou et al. 2019; Chery et al. 2020). Intact PCMs exhibited a lower elastic modulus compared to PCMs that were depleted of perlecan and were mainly composed of collagen type VI (Wilusz et al. 2012). However, a lack of collagen type VI also decreases the PCM mechanical properties (Alexopoulos et al. 2009; Zelenski et al. 2015; Chery et al. 2020).

These changes in PCM mechanical properties directly influence the chondrocytes. Several studies have shown that a loss of the PCM or a decreased PG content in the PCM leads to increased cell deformation (Millward-Sadler et al. 2004) (Guilak et al. 2006; Danalache et al. 2019; Chery et al. 2020). Recent *in silico studies* have revealed that the softening of the PCM resulted in an increase of strain in the PCM as well as the chondrocyte, even when the macroscale mechanical properties of the tissue did not change (Khoshgoftar et al. 2017). This highlights the importance of the PCM in attenuating mechanical stress and strain shielding of the chondrocytes especially in the superficial zone of articular cartilage where stresses are higher. In a study subjecting bovine articular cartilage explants to hyperphysiological impact loading resulting in cell death due to high strain on integrins. However, when the cell-matrix adhesion was inhibited the cells did not die under injury loading (Sauter et al. 2012; Jang et al. 2014).

Mechanical integrity of the PCM is necessary for physiological mechanotransduction and cartilage health. It is crucial in providing the right mechanical cues for chondrocytes homeostasis and is imperative for the cell-matrix connection. A change in mechanical properties of the PCM seems to have a more direct influence on the cells than bulk tissue strain. It is however not well understood how the heterogeneous strain from the tissue is received by the PCM on the microscale and how this impacts the cells.

### Chondrocytes

There is evidence that the nonuniform ECM and resulting strain transfer through the tissue influences chondrocyte deformation and morphology. Cell shape is influenced by the ECM composition which is different in the different zones (Figure 1) (Benya 1988; Guilak, 1995). Chondrocytes in the superficial zone, where there is a lower compressive modulus, experience larger changes in volume compared to chondrocytes in the other zones (Mansfield et al. 2015). There can be a wide range of changes in cell thickness in the same zone under compression, indicating that even in the same zone, cells might not experience the same mechanical load (Mansfield et al. 2015; Kwon et al. 2016). Under compression no significant chondrocyte shape changes or displacement were detected in the plane parallel to the surface, whereas under tensile load, all chondrocytes deformed parallel to the articular surface. At a tissue strain of 38%, the median compressive strain in the chondrocytes was 46% (Mansfield et al. 2015). These deformation differences could be due to the original shape of the chondrocytes before mechanical stimulation. In meniscal cartilage, spherical cells deform less than elongated cells which readily deform (Han et al. 2013). Besides *in vitro* experiments, different *in silico* models have highlighted this relationship between cellular deformation and tissue heterogeneity (Breuls et al. 2002; Klika et al. 2019; Tanska et al. 2020).

In addition to variable changes in cell morphology, intracellular strains are also nonuniform. Chondrocyte deformation generates heterogeneous intracellular strain fields which appear to be dependent on cell organelle organisation (Knight et al. 2006). Cell nuclei deform less than the cytoplasm as they have a higher stiffness (Gilchrist et al. 2007). Chondrocyte metabolism is influenced by ECM composition and zonal arrangement (Benya 1988; Guilak et al. 1995; Hall 2019). Elevated strain magnitudes, especially in the superficial zone of articular cartilage, are associated with mitochondrial dysfunction, apoptosis, and cell death (Bonnevie et al. 2018). Although this demonstrates that the mechanical environment influences chondrocyte metabolism, it remains unknown what effects this has in the long term and how this heterogeneous strain affects tissue remodelling over time. Furthermore, it is unclear how stress and strain reach cells and are distributed through the whole heterogeneous tissue, and further how a change in matrix composition and integrity influences the forces sensed in the microenvironment of the cell.

## Further Directions and Outlook

The transfer of stress and strain signals to and from cells in tissues involves the integration of the ECM. Different cartilage types have a distinct overall ECM molecular composition, that is spatially highly heterogeneous. The heterogeneity in the ratio of ECM molecules is different in cartilage tissues and regions and leads to a depth-dependent non-uniform compressive strain transfer and alters the magnitude of forces sensed by cells in articular and fibrocartilage, influencing chondrocyte metabolism and biochemical response.

As our understanding of how strain and force are transferred through cartilage tissue grows, it is evident that it is a multiscale process. The influence of matrix molecule interaction and distribution is not well understood, and even though single cell responses to mechanical stimuli are well studied, there is a lack of understanding of the long term effects. It will be important to link the cell response to the tissue scale forces and longitudinally investigate remodelling over a longer time span.

Investigation of multiscale strain transfer would further our understanding of the complex mechanisms of mechanobiology and remodelling in different cartilage tissues, not only load bearing types. Furthermore, it would increase our knowledge about native cartilage tissue, providing a benchmark by which to compare tissue engineered constructs and feed into the development of effective treatment strategies to address cartilage pathologies.
